# A wearable real-time kinetic measurement sensor setup for human locomotion

**DOI:** 10.1017/wtc.2023.7

**Published:** 2023-04-11

**Authors:** Huawei Wang, Akash Basu, Guillaume Durandau, Massimo Sartori

**Affiliations:** 1 Department of Biomechanical Engineering, University of Twente, Enschede, The Netherlands; 2 Department of Mechanical Engineering, McGill University, Montreal, QC, Canada

**Keywords:** biomechanics, biomechatronics, monitors, sensors

## Abstract

Current laboratory-based setups (optical marker cameras + force plates) for human motion measurement require participants to stay in a constrained capture region which forbids rich movement types. This study established a fully wearable system, based on commercially available sensors (inertial measurement units + pressure insoles), that can measure both kinematic and kinetic motion data simultaneously and support wireless frame-by-frame streaming. In addition, its capability and accuracy were tested against a conventional laboratory-based setup. An experiment was conducted, with 9 participants wearing the wearable measurement system and performing 13 daily motion activities, from slow walking to fast running, together with vertical jump, squat, lunge, and single-leg landing, inside the capture space of the laboratory-based motion capture system. The recorded sensor data were post-processed to obtain joint angles, ground reaction forces (GRFs), and joint torques (via multi-body inverse dynamics). Compared to the laboratory-based system, the established wearable measurement system can measure accurate information of all lower limb joint angles (Pearson’s *r* = 0.929), vertical GRFs (Pearson’s *r* = 0.954), and ankle joint torques (Pearson’s *r* = 0.917). Center of pressure (CoP) in the anterior–posterior direction and knee joint torques were fairly matched (Pearson’s *r* = 0.683 and 0.612, respectively). Calculated hip joint torques and measured medial–lateral CoP did not match with the laboratory-based system (Pearson’s *r* = 0.21 and 0.47, respectively). Furthermore, both raw and processed datasets are openly accessible (https://doi.org/10.5281/zenodo.6457662). Documentation, data processing codes, and guidelines to establish the real-time wearable kinetic measurement system are also shared (https://github.com/HuaweiWang/WearableMeasurementSystem).

## 1. Introduction

The development of systems for the precise quantitative measurements of body kinematics and kinetics has enabled advanced movement analyses (Hewett et al., 2005; Hof, 2008; Marcela and Diaz, 2009; Winter, 2009; Enoka, 2015; Wang, 2020; Keemink et al., 2021). Established measurement systems based on optical cameras and ground reaction force (GRF) plates (Richards, 1999; Moore et al., 2015; Camargo et al., 2021) provide consistent and accurate recordings of human body movements across many skeletal degrees of freedom (DoFs). However, they require participants to move within highly controlled and constrained volumes that do not reflect the real world we live and move in. Moreover, the associated high costs further limit their applicability and translation to a variety of settings, for example, from clinical to occupational. Wearable and untethered measurement systems are promising solutions to enable out-of-lab analyses. However, an easily accessible setup that can support real-time kinetic measurement has not been proposed and thoroughly validated yet.

Inertial measurement units (IMUs) and pressure insoles are two major types of wearable sensors that have been studied and developed in laboratories to capture body kinematics and GRF surrogates (Liu et al., 2012; Jacobs and Ferris, 2015; Kim et al., 2017; Faber et al., 2018; Fukushi et al., 2019; Cui et al., 2021). However, compared to well tested and compactly designed commercial sensors (Moticon, Germany; Nansense, the United States; Pedar, Germany; Rokoko, Denmark; Tekscan, the United States), in-lab developed systems are mostly manually manufactured, which are far from being widely available to other research groups and individuals. As a result, more and more biomechanical and clinical studies have employed the commonly available commercial sensors to achieve their goal of measurement and assessment (Cutti et al., 2008; van den Noort et al., 2009; Pau et al., 2014; Wannop et al., 2020; Wouda et al., 2021; Fereydounnia et al., 2022; He et al., 2022; Trkov et al., 2022). These studies nevertheless largely focus on one type of sensors, in isolation, which can only provide indications on how good they are in measuring a sub-set of the human movement data and as a result cannot support the internal biomechanical states estimation (Ferrari et al., 2010; Zhang et al., 2013; Braun et al., 2015; Stöggl and Martine, 2016; Oerbekke et al., 2017; Al-Amri et al., 2018; Price, 2018). Specifically, estimating joint torques via multi-body inverse dynamics requires not only body postures but also the external forces (GRFs) (Hatze, 2002). Several studies tried to use both commercial IMUs and pressure insole sensors together in movement analyses (Benocci et al., 2009; Abdelhady et al., 2019; Wang et al., 2019; Matsumura et al., 2021). Data of joint angles, pressure distributions, and gait/event detections were extracted. However, joint torque analyses were not performed by combining those extracted information together. Joint torques are critical intermediate-layer information that connect visible movement appearances (joint angles) with underlying internal neuromuscular systems (muscle states and forces) (Heintz and Gutierrez-Farewik, 2007; Liu et al., 2008; Afschrift et al., 2021; Moya-Esteban et al., 2022; Simonetti et al., 2022) and thus are highly desired to be estimated in biomechanical measurements. To the best of our knowledge, only one study was done in the past which calculated joint torques through multi-body inverse dynamics by utilizing both IMUs and pressure insoles and compared its results with a laboratory-based measurement setup (Khurelbaatar et al., 2015). However, only one slow walking condition was tested, and a very low number of participants were recruited.

Efforts have also been spent to explore other ways of extracting joint torques from wearable measurement sensors; however, their results are limited and ungeneralizable. Body kinetics (joint torques) and even muscular information have been estimated by using solely IMU sensors (Karatsidis et al., 2016; Wouda et al., 2018; Tanghe et al., 2019; Dorschky et al., 2020; Kim et al., 2020; Matijevich et al., 2020), for which either machine learning or probabilistic methods were used. These methods require a wide range of training data and are hard to extrapolate to new scenarios (Gurchiek et al., 2019). One recent study by Karatsidis et al (2019) tried to solve this issue with extrapolation, by using a musculoskeletal model in which effort is minimized (Fluit et al., 2014). They introduced a ground contact model and estimated fully body kinetics and muscle forces from kinematic data only. However, this method is computationally expensive, especially for lengthy measurements. In addition, the accuracy of ground contact models, which determines how good the estimation of joint torques are, can be an issue, especially for complex ground scenarios, such as uneven terrains.

Considering current missing elements in wearable measurement systems from the state of the art, in this study we focused on establishing a commercial sensor-based (easily accessible), fully wearable (wireless data transfer), real-time (frame-by-frame streaming) kinetic measurement setup and validated it at the joint kinetic (torque) level among a large range of movement types, against the conventional laboratory setups. Compared to previous studies and apart from the direct features provided by commercial sensors, our unique contributions are

- Identifying suitable fully wearable sensors for the real-time kinetic measurement system.
- Establishing the synchronized, real-time frame-by-frame data streaming protocol.^1^
- Systematically validating the wearable sensor setup at the joint kinetic level against to the current golden-standard conventional system with rich movement types.^2^
- Identifying the issues and error sources of the wearable sensor setup and indicating the future directions of improvement.
- Fully open sourcing the validation data and codes, so that researchers can contribute to the future work together.^3^

## 2. Methods

### 2.1. Experiment setup

Nine healthy volunteers (age: 25.4 ± 2.7, height: 1.73 ± 0.08 m, weight: 68.1 ± 9.0 kg) with no self-reported history of neurological or musculoskeletal impairments participated in this study. The Natural Sciences and Engineering Sciences Ethics Committee of the University of Twente approved the experimental procedures (reference number: 2021.57), and all participants provided written informed consent. The authors assert that all procedures contributing to this work comply with the ethical standards of the relevant national and institutional committees on human experimentation and with the Helsinki Declaration of 1975, as revised in 2008. General information about participants can be found in Table 1.

**Table 1.** Participants information

Participant no.	Height (m)	Weight (kg)	Age (years)	Foot size (EU)	Gender (F/M)
P04	1.80	75.2	23	43	M
P05	1.71	65.1	23	39	F
P06	1.68	56	23	39	F
P07	1.82	80.8	23	43	M
P08	1.62	61.5	28	39	F
P09	1.82	81	26	43	M
P10	1.66	61.6	28	43	M
P11	1.68	62.4	25	39	F
P12	1.79	69	30	42	M

*Note.* Participant numbers start from 04, since the first three participants were excluded due to unmatured testing protocol.

**Figure 1.**
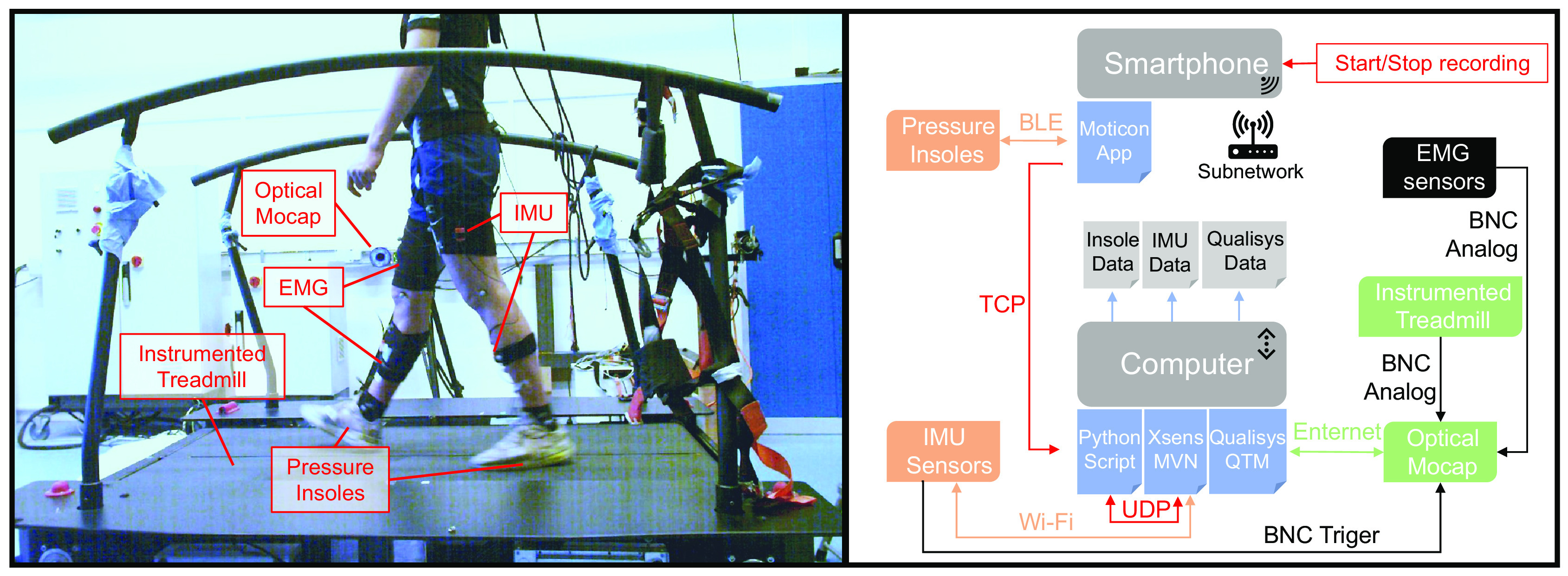
Experimental setup (left) and sensor hardware synchronization architecture (right). The pressure insoles and the IMU sensors (in orange color) form the wearable system. The optical mocap and the instrumented treadmill (in green color) form the laboratory-based system. EMG sensors were indicated with black color, since they can belong to either side.

The experimental setup is shown in Figure 1 (left). Six types of measurement devices were used to capture different information about participants’ movements. They can be divided into two systems: the wearable system and the lab setup. In the wearable measurement system, eight IMUs (Xsens Link, Enschede, The Netherlands) were used to measure the kinematics of the lower limbs and trunk. A pair of pressure insoles (Moticon, Munich, Germany) was used to measure vertical GRFs as well as anterior–posterior and medial–lateral center of pressure (CoP) trajectories. In the laboratory-based setup, an optical motion capture (OMC) system containing eight infrared light cameras (Oqus 6+ series, Qualisys, Gothenburg, Sweden) was used to measure body kinematics using reflective markers. A split-belt instrumented treadmill (Motek-Forcelink BV, Culemborg, The Netherlands) was used to measure the full DoFs GRFs under each foot. Two video cameras were also included inside the laboratory-based system to capture RGB images of participants’ body postures in the sagittal and frontal planes (Miqus video, Qualisys, Gothenburg, Sweden). In addition, nine electromyography sensors (EMGs) (Delsys Trigno, Delsys, USA) were included to record the activations of nine major muscles in the dominant leg.

A synchronization protocol was developed to start and stop the recording of all six types of measurement sensors at the same time (Figure 1 (right)). As shown in Figure 1, a smartphone and a computer were used as the main hosting devices that interacted with the above-mentioned devices. They were connected to the same subnetwork (generated by the Xsens WiFi router). The Moticon application (App) that was installed on the smartphone was used to communicate and control the Moticon insoles through the low-energy Bluetooth protocol (Vilzmann, 2021). The Xsens MVN software installed on the computer was used to communicate with the Xsens IMU sensors. The Qualisys QTM software installed on the same computer was used to connect and control the infrared cameras and two video cameras. The instrumented treadmill and the EMG sensors were connected to the Qualisys system through a 64-channel analog to digital converter. A Python script was written and ran on the hosting computer to establish the transmission control protocol (TCP) communication with the Moticon App and user datagram protocol (UDP) communication with the Xsens MVN software, respectively. A Bayonet Nut Coupling (BNC) cable was connected between the Xsens trigger module and the OMC.

The start and stop recording commands were originated from the Moticon App, and all sensors were started and stopped in the following order: The start/stop commands were first sent to the Moticon insoles to start or stop their recordings. The Python script ran on the computer was in idle mode and waiting to detect the data flow sent by the Moticon insoles through TCP. Once it detected the data, the script sent a starting command to the Xsens MVN software to start its recording through its UDP remote control feature. Once the Xsens MVN software started recording data, a trigger signal was sent to the Qualisys system through the BNC cable to start its recording. In the meantime, data of the EMG sensors and the instrumented treadmill were simultaneously streamed inside the Qualisys QTM software via analog channels. Correspondingly, the script stopped the recording of all sensors once it detected the stop of pressure insole data. At the end of each recording, an insole data file (.txt), an IMU data file (.mvn), and a Qualisys data file (.qtm) were saved that contain all six sensors’ data.

### 2.2. Experimental procedures

Before each participant’s recording, the Qualisys OMC system was calibrated following the recommended procedure. Offsets of the instrumented treadmill load cells, with no weight on it, were removed to reduce measurement artifacts. Then, participants’ body weight, body height, and segment lengths were measured for the virtual avatar personalization inside the Xsens MVN software and the Moticon App. Afterwards, eight IMU sensors were placed on the participants’ legs, pelvis, and trunk using straps, according to the lower body with sternum setup. Pressure insoles were put inside participants’ shoes at the same stage. This was followed by the default calibration procedure of the Xsens MVN software and the Moticon App. Nine wireless EMG sensors were then placed at the dominant leg to capture activations of the following muscles: soleus, medial gastrocnemius, lateral gastrocnemius, tibialis anterior, semimembranosus, biceps femoris long head, vastus lateral, rectus femoris, and vastus medial, according to the SENIAM guidelines. Hair at the EMG measuring locations was shaved, and skin was cleaned using a 95% alcohol water solution. Last, 33 reflective markers were placed on participants’ lower limbs and the trunk using the same protocol as previously presented (Durandau et al., 2018).

Participants were then asked to stand on the treadmill, and a 10-second static standing trial was recorded for scaling the OpenSim gait model used for post-processing. A maximal voluntary contraction was recorded with participants doing the following four movements using their muscles as hard as possible: calf raise, toe up, kicking backward, and kicking forward. In the kicking back and kicking forward movements, one experimenter held the ankle of participants to provide resistance. Afterwards, participants were asked to perform six walking trials (0.9, 1.8, 2.7, 3.6, 4.5, 5.4 km/h) and three running trials (6.3, 8.8, 9.9 km/h). Each trial lasted 1 minute. Four non-locomotion trials (vertical jump, squat, lunge, and single-leg landing) were performed at the end with 10 repetitions of each movement type. Participants were free to ask for a rest whenever they wanted to; however, no participant asked, which indicates that their muscles were not in a heavy fatigued state during the recordings.

### 2.3. Movement data processing

**Figure 2.**
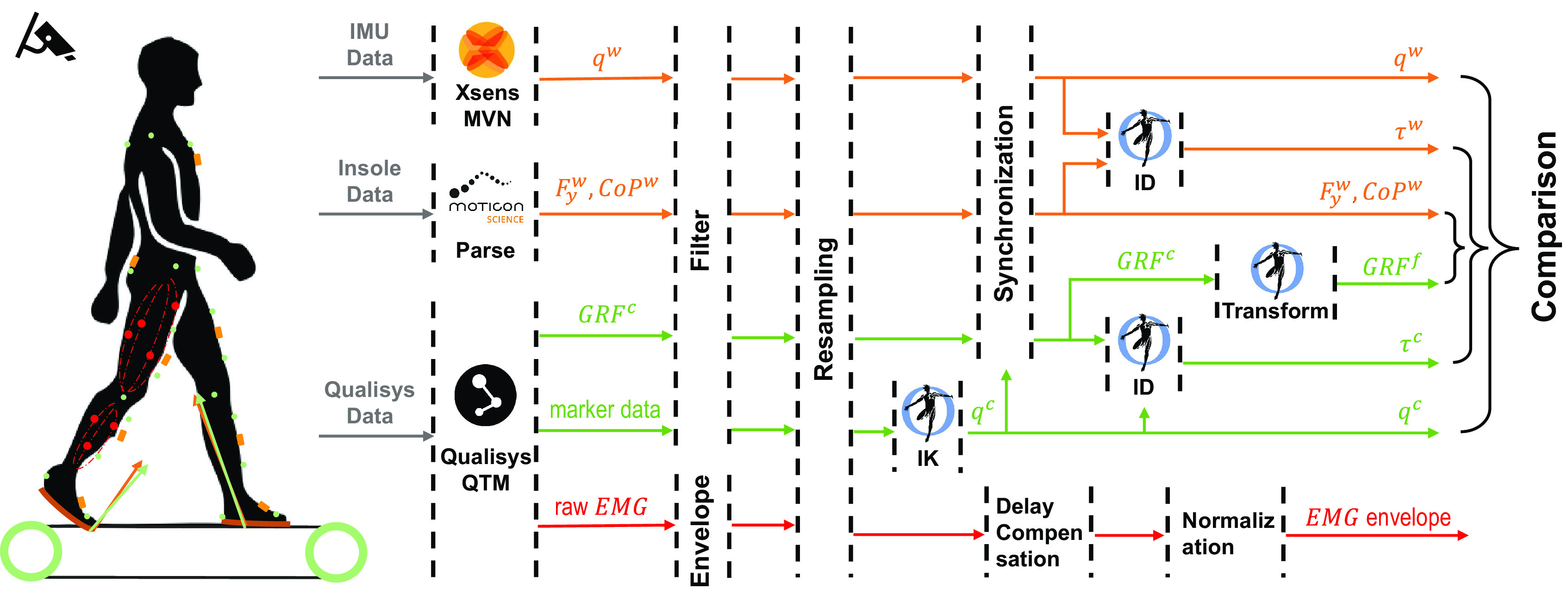
Data processing pipeline. The processing arrows in orange color are for the wearable measurement system. The processing arrows in green color are for the laboratory-based measurement system. The red arrows are the EMG processing steps.

Data were processed using customized Matlab (Matlab 2019b, Mathworks) scripts. The major processing steps included (Figure 2): low-pass filtering (2nd order Butterworth filtfilt, 12 Hz cutoff; Kristianslund et al., 2012) the raw sensor data; resampling all the sensor data with the same rate (100 Hz); inverse kinematics through the marker data for extracting joint angles from the laboratory-based system using the OpenSim tool (Delp et al., 2007); and offline synchronization of the kinematic and vertical GRF data between the wearable and laboratory-based systems. After synchronization, inverse dynamics (from OpenSim tool) were applied to both the wearable system and the laboratory-based system to calculate joint torques; wireless transfer delay was compensated for the EMG recordings, and normalized activation envelopes were extracted, based on the recommended processing steps (Konrad, 2005). Furthermore, GRFs measured by the treadmill were transformed from the Qualisys global coordinate frame to the calcaneus coordinate frame of each foot via OpenSim functions (Delp et al., 2007) to enable fair comparisons with the pressure insole measurements. Gait cycles were separated using the vertical GRF by detecting heel strike for walking and running trials, with a threshold of 50 N and 100 N, respectively (a trade-off between the accurate heel-strike data point and the miss-detection ratio). For the four non-locomotion tasks (vertical jump, squat, lunge, and single-leg landing), starting and ending frames of each motion repetition were manually detected using the GRF under each foot.

### 2.4. Multi-body dynamic model

The OpenSim gait2392 model (Delp et al., 2007) was used to perform the inverse kinematics and inverse dynamics analyses mentioned in Section 2.3. For each participant, this original model was first scaled, based on the static standing marker data. Averaged joint angles from the Xsens MVN software of the same static trial were used as references to avoid unreasonable scaling outcomes. Then, the scaled model was used for the inverse kinematics calculations of the other trials, with all markers’ weights put to 1. Lastly, the calculated joint angles were used together with the full GRF measured from the instrumented treadmill to compute joint torques via the OpenSim inverse dynamics tool. The same scaled OpenSim model was used to compute joint torques for the wearable system also. Joint angles from the Xsens MVN were mapped to the OpenSim model according to the coordinate definitions. Vertical GRFs and CoPs from the pressure insoles were applied to the OpenSim model with the assumption that the most rear point of the pressure insole is aligned with the coordinate origin of the calcaneus bone.

### 2.5. Evaluation procedures

Comparisons between the wearable system and the laboratory-based system were done at both kinematic and kinetic levels. Several tests were applied on each participant’s data to evaluate them thoroughly. The first test assessed the similarities of joint angles estimated from the two systems. Curve similarities of the motion tasks were quantified by the Pearson’s correlation coefficients (PCCs) (value changes between −1 and 1). PCC number of 1 represents the highest similarity of the variable change trends (exactly the same). Number of 0 represents no correlation at all. PCC value of −1 means the exact opposite direction. Quantitative errors between these two measurement systems were indicated by computing the root mean squared errors (RMSE). Unnormalized joint angle values were used for these two calculations. Test 2 evaluated the similarities and quantitative errors for the vertical GRFs and CoPs. Similarly, the PCC and RMSE were calculated. When calculating the RMSE, vertical GRFs were normalized by participant body weight, and CoPs were normalized by pressure insole length (L) and width (W), in the posterior–anterior and medial–lateral directions, respectively. Lastly, the PCC and the RMSE were calculated for the joint torque comparisons. Joint torques were normalized by each participant’s body mass. Finally, PCC and RMSE were averaged among all participants and all movement trials to provide an overall assessment of the wearable system against the laboratory-based setup.

## 3. Results

Kinematic and kinetic data of both measurement systems were calculated in the 3D space. However, only the sagittal plane (and the CoP lateral) results are shown here, as they covered the major movements of tested motion types. Results of the other anatomical planes can be found in the shared dataset (10.5281/zenodo.6457662). PCC and RMSE of three leg joints (hip flexion/extension, knee flexion/extension, ankle plantar/dorsiflexion) are shown first to provide quantitative evaluation of the wearable measurement system against the laboratory-based setup. In addition, phase plots of the kinematic and kinetic variables of three tested movements (a middle-speed walking, a middle-speed running, and a lunge movement) are shown to demonstrate the detailed variable similarities and differences along the movement phases, between the two measurement systems. Plots of other tested motion tasks can be found in Appendix B. Finally, summary plots of the open-access dataset are provided.

### 3.1. Quantitative evaluations

**Figure 3.**
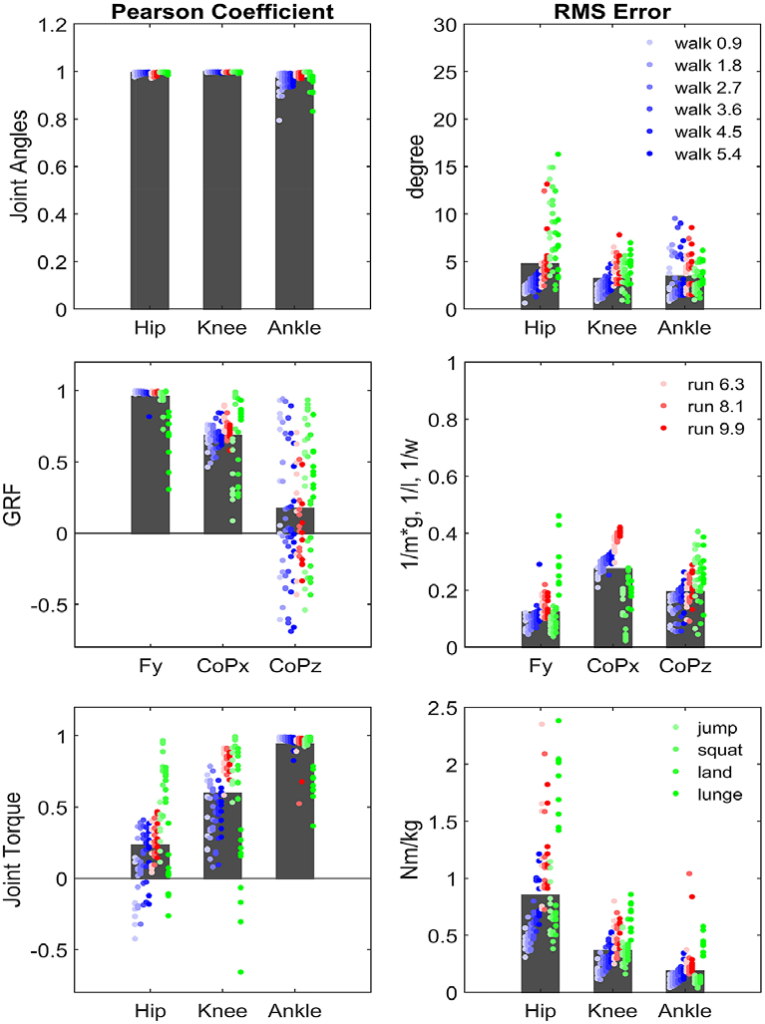
PCC and RMSE of joint angles, GRFs, and joint torques. Values of the bar plots are the averaged results among all participants and all tested motion trials. Colored dots (with different amounts of fading) indicate the PCC and RMSE for each participant for each motion type. Results for the same motion type are plotted in the same column and different dots represent different participants. Numbers in the legends indicate the gait speed in km/h.

PCC and RMSE were calculated to quantitatively compare the two measurement systems for all participants and all tested motion types (Figure 3). As shown in the first row (results of Test 1), joint angles for all three joints showed very high correlation (*r* = 0.929 on average) and low RMSE (3–5° on average), between the wearable and laboratory-based systems. Results of the second test are shown in the second row. Vertical GRFs presented high correlations (*r* = 0.948 on average) and low RMSE (12% of the body weight), except for the lunge movement (green dots in the rightest column). CoP in the anterior–posterior (walking) direction (CoPx) showed a fair match with the laboratory-based setup (*r* = 0.69, RMSE = 0.27 foot length). Among the tested motion types, running trials have the largest RMSE (red dots), followed by the walking trials (blue dots). RMSE of non-locomotion trials (green dots) have the lowest values. The CoP measured by the wearable system, in the medial–lateral direction (CoPz), showed no similarity at all with the laboratory-based measurements (*r* = 0.17 on average) but has relatively low absolute errors (RMSE = 0.18 foot width). Joint torque comparisons (Test 3) are shown in the last row of subplots, the correlation of ankle joint torques indicated a good match (*r* = 0.931), and low average RMSE are shown (0.20 Nm/kg), except for two running trials (might be outliers) and the lunge movement. Knee joint torques showed fair matches (*r* = 0.61, RMSE = 0.37 Nm/kg), whereas, for slow walking and lunge movements, low correlations are shown (blue dots and green dots). Hip joint torques showed very little correlation and large RMSE (*r* = 0.23, RMSE = 0.85 Nm/kg), indicating that the wearable system cannot support accurate hip joint torque calculations via multi-body inverse dynamics.

Results among the tested motion types (the faded blue, red, and green dots in Figure 3) show a clear trend also. The absolute errors of joint angles, especially at the hip and knee joints, gradually increased along with the increase of locomotion speed and for the four non-locomotion movements (jump, squat, lunge, land), partially due to the larger joint motion ranges of these movements. This indicates that the joint angle errors of the wearable measurement system are proportional to the motion ranges, instead of being constant. Similar effects are seen in the GRF measurements. RMSE of vertical GRF force and CoPx gradually increased from slow walking to fast walking and dropped for the four non-locomotion tasks. Given the fact that these variables have the largest and most rapid changes during fast running movements, this trend indicates that the GRF errors in the wearable measurement system were also proportional to the amplitude. Since joint torques were calculated from the joint angles and GRFs using inverse dynamics, they share similar error trends with the ones mentioned in the joint angles and GRFs measurements.

### 3.2. Variable phase plots

**Figure 4.**
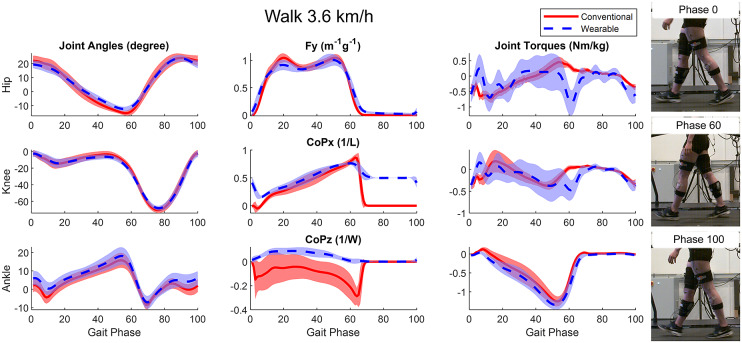
Averaged joint angles (first column), GRFs (second column), and joint torques (third column) in the sagittal plane calculated from the laboratory-based and wearable measurement systems of all participants at a walking speed of 3.6 km/h. The Fy, CoPx, and CoPz were transformed into the calcaneus coordinate frame of the scaled OpenSim model for each participant. Red solid lines and blue dashed lines represent the mean value of these variables for the laboratory-based and wearable systems, respectively. The shaded areas indicate ± a single standard deviation of the corresponding variables. The participant in the demonstrating video frames has their right leg as the dominant side.

**Figure 5.**
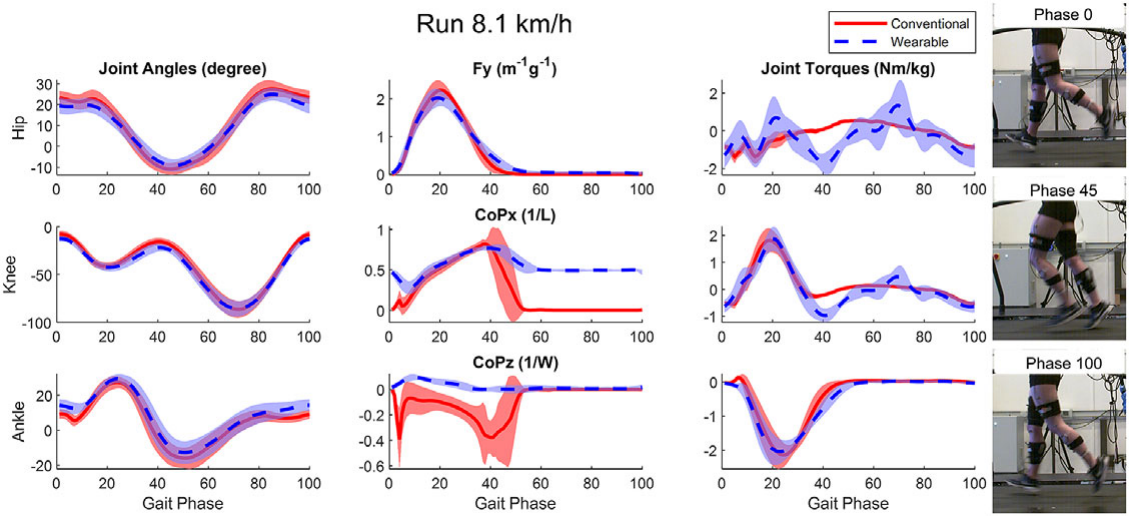
Averaged joint angles (first column), GRFs (second column), and joint torques (third column) in the sagittal plane calculated from the laboratory-based and wearable measurement systems of all participants at a running speed of 8.1 km/h. The Fy, CoPx, and CoPz were transformed into the calcaneus coordinate frame of the scaled OpenSim model for each participant. Red solid lines and blue dashed lines represent the mean value of these variables for the laboratory-based and wearable systems, respectively. The shaded areas indicate ± a single standard deviation of the corresponding variables. The participant in the demonstrating video frames has their right leg as the dominant side.

**Figure 6.**
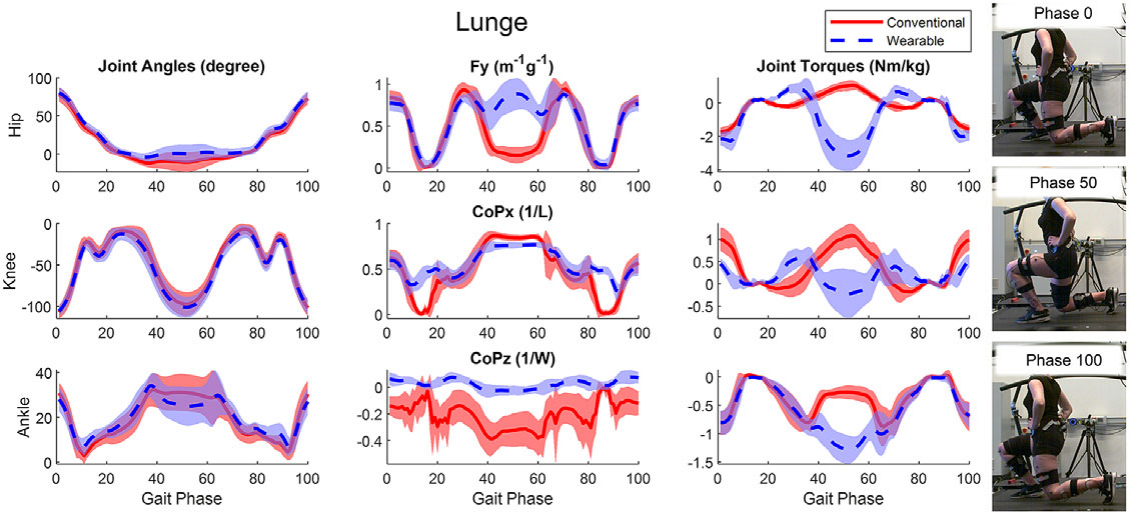
Averaged joint angles (first column), GRFs (second column), and joint torques (third column) in the sagittal plane calculated from the laboratory-based and wearable measurement systems of all participants at the lunge movement. The Fy, CoPx, and CoPz were transformed into the calcaneus coordinate frame of the scaled OpenSim model for each participant. Red solid lines and blue dashed lines represent the mean value of these variables for the laboratory-based and wearable systems, respectively. The shaded areas indicate ± a single standard deviation of the corresponding variables. The participant in the demonstrating video frames has their right leg as the dominant side.

Data of walking at 3.6 km/h, running at 8.1 km/h, and the lunge movement trials are plotted in Figures 4–6, respectively, to demonstrate the difference within each variable during different movement phases. The same variables as shown in Section 3.1 are shown here. These figures represent the averaged data of repeated movement cycles of all participants for the same motion type. As indicated with high PCC and low RMSE, joint angle phase plots showed close matches between wearable and laboratory-based systems, among all three selected motion types.

In both measurement systems, the vertical GRFs (Fy, top plot in the second column) during walking had similar smooth ‘M’ shapes and showed a similar ‘n’ shape during running, whereas, in the lunge movement, vertical force measured by the wearable system has a large difference (around 0.8 body weight) compared to the laboratory-based system at the midpoint (50%) of the movement, where participants supported themselves with the toe of the dominant leg. In the measurement of the laboratory-based system, CoPx (anterior–posterior direction) curves raised from around 0 to nearly 1 indicating that the CoP gradually shifted from heel to toe in both walking and running. However, the wearable measurement system captured different trajectories of the CoPx: from heel strike, it started from the middle part of the foot and then gradually moved toward the heel, and before reaching the heel, changed the movement direction to the frontal part of the foot. The CoPz (medial–lateral direction) measurements in these two systems have large differences. In the laboratory-based measurement system, the CoPz is mostly located on the medial side of the foot (0 value is the middle, positive values are in the lateral direction). There are two quick and large shifts toward the medial direction at the beginning and end of the stance phases. Whereas, in the wearable system, the CoPz is slightly on the lateral side at all times. No medial shifts were measured during walking and running. Standard deviations of the CoPz in the laboratory-based system are also larger than the wearable measurements.

In terms of joint torques, in the walking and running trials, joint torques of the ankle and knee that are estimated with the wearable measurement system are close to the ones from the laboratory-based system. However, hip joint torque presents significant differences. For lunge movements, due to the differences in GRF’s vertical force in the middle of the movement phases, all three joint torques from the wearable system have large differences, compared to the laboratory-based system. Apart from the middle phases, ankle and hip joints show good matches, whereas the knee joint torques present larger differences.

### 3.3. Open-access dataset

**Figure 7.**
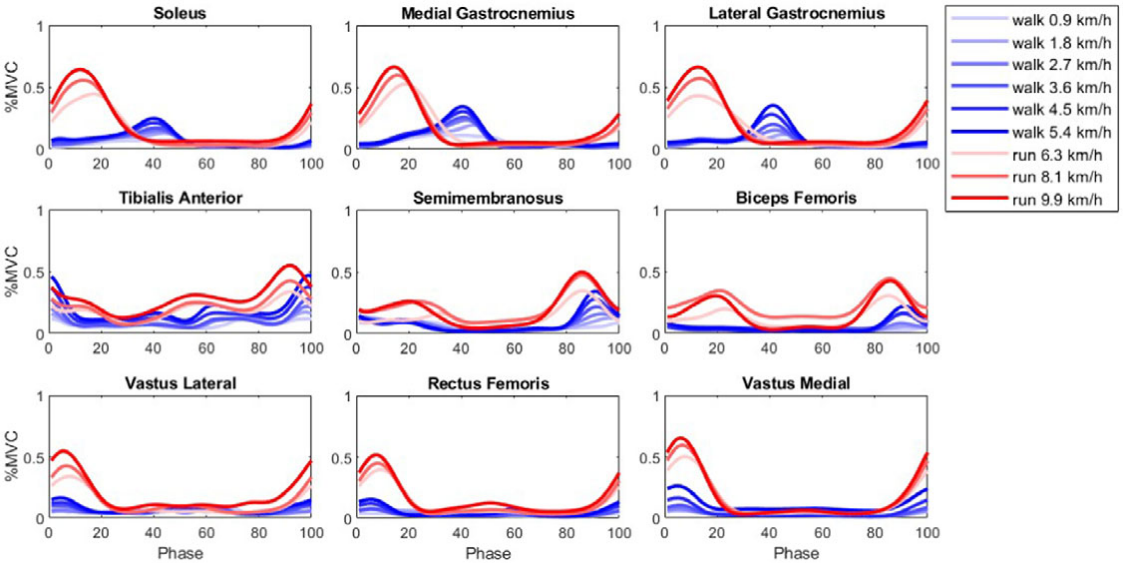
The averaged nine muscle activation envelopes of all gait cycles and all participants at different walking and running speeds. The walking trials were plotted in blue. The running trials were plotted in red. The shade level of the lines indicates the changes in locomotion speeds.

**Figure 8.**
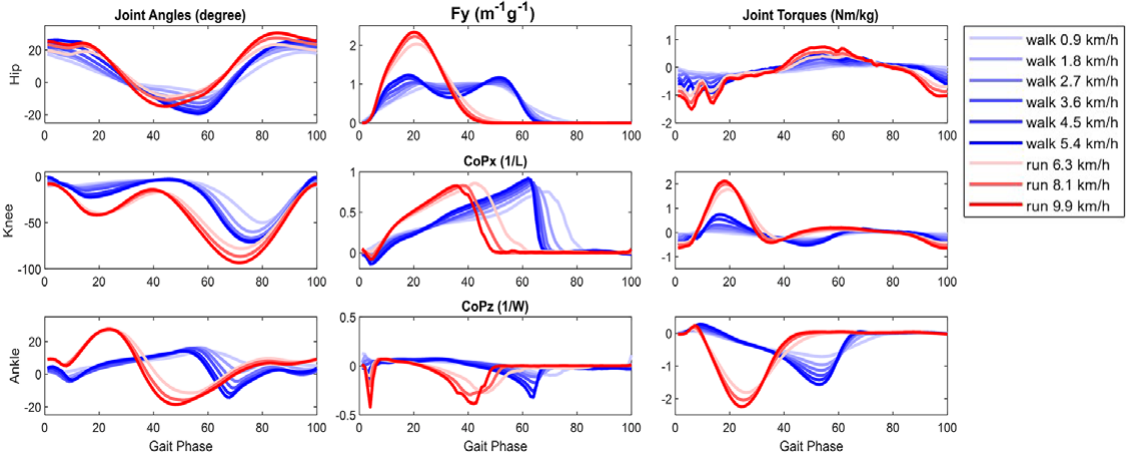
Averaged Joint angles, GRFs, and joint torques in the sagittal plane calculated from the wearable measurement system for all participants. The walking trials were plotted as the blue line with the shade indicating the speed. Correspondingly, the running trials were plotted in red.

A comprehensive dataset was generated from this study which contains 6 types of synchronized sensors (including EMGs, Figure 7), 12 participants, and 13 motion trials each. Both raw and processed data are included in the dataset. The post-processing code is also provided to increase the usability of this dataset for different research targets, in which users can adjust or extend the provided post-processing code. An open repository that contains code and documentation to fully reproduce the studied wearable measurement system is also included.^3^ A summary plot of the kinetic data of the locomotion tasks measured with the wearable measurement system is shown in Figure 8. In both walking and running trials, joint angles, GRFs, and joint torques all had reasonable gradual changes along with the changes in speed.

## 4. Discussion

In this work, we established a wearable kinetic measurement sensor setup based on commercially available IMUs and pressure insoles. Its capability and accuracy were validated against a laboratory-based measurement system. Joint angles, GRFs, and joint torques that were measured by the laboratory-based system provide joint kinetic profiles in line with previous studies (Mann and Hagy, 1980; Novacheck, 1998; Uchida and Delp, 2021). We open sourced all the code as well as the recorded dataset. In the dataset, both raw and processed measurements were provided. Even though we used commercial sensors, like Xsens IMUs, the dataset we provided can be directly used without requiring commercial software or licenses. The recorded IMU data included raw accelerations and magnetic fields but without raw angular velocities, due to the missing exporting feature of the Xsens MVN software. This missing raw information will be included once the new exporting feature is added in the future software update.

The proposed IMU sensors provided accurate joint angle measurements compared to the OMC system, in the walking and running trials. However, in the other four non-locomotion tasks (vertical jump, squat, lunge, and single-leg landing), the hip flexion angle measurements were less accurate (green dots in the top right subplot of Figure 3). This might be caused by the fact that these motions require much larger hip and pelvis motions. Large pelvis tilt movements can cause movement of the pelvis IMU sensor with respect to the pelvis body segment, because the Xsens body straps were used to fix the IMU sensors to body segments. Taping the sensors directly on the pelvis might generate better measurements for the non-locomotion tasks.

One significant difference in vertical GRFs, between the two measurement systems, is the CoPx at the early stance and swing phases. This difference is due to the assumption of the used pressure insoles that the CoPx is in the middle of the feet when no force is detected (swing phases). Therefore, when heel strike happened, the CoPx detected by insoles started shifting from mid-foot to heel. Whereas changing rates of CoPs in pressure insoles were also limited, the CoPx did not immediately shift to the heel but moved toward it with a negative slope, until it met with the actual CoPx, which was moving toward the toe from the heel. These “v”-shaped curves in pressure insole CoPx measurements also caused the issue that almost no ankle plantarflexion torques were captured by the wearable measurement system (bottom right in Figure 4). Given these results, we would suggest manufacturers to change unloaded default location of CoPx from middle of the foot to the heel and increase limits of changing rates in CoPs, contingent to measurement noise acceptability.

The measured GRFs have bad fits in the lunges, among all tested motion types. One major reason might be that the lunge movement requires standing on one’s toes. This is not a valid usage scenario for the tested pressure insoles, especially because they are quite stiff. This means that they could not measure precise GRFs for movements that require standing on one’s toes. In addition, in almost all movement types we recorded, static force differences existed in measured vertical GRF, even though the pressure insoles were calibrated using each participant’s body weight. This may be due to the current calibration process that only contains dynamic movements, such as slow walking and weight-shifting between 2 feet. Even though the total body weight is a known value (input by users), in dynamic movements, the force profiles under each foot depend on how the movements are performed. For instance, different users have different speeds of slow walking. Different walking speeds will result in different GRF shapes. Using one criterion to calibrate these dynamic movements might cause errors for different users. We suggest including static tasks, such as single-leg static standing, in the calibration process to increase the calibration accuracy.

Torques at the knee and hip joints that were calculated using the wearable system had large errors compared to the laboratory-based measurement system. The major reason is that no shear forces were measured in the wearable system. Missing shear forces have larger effects on the proximal joints (hip) than on the distal joints (ankle), simply due to the increase of moment arms (approximately the height of the joint with respect to the ground). The horizontal forces were not estimated in the current work since it is a very complex problem. Studies have tried to use either machine learning or dynamic model-based methods (Sartori et al., 2013; Jung et al., 2014; Karatsidis et al., 2016; Bastien et al., 2019; Hendry et al., 2020; Refai et al., 2020). However, neither method is generic. Machine learning methods largely rely on the training dataset and have extrapolation issues, while dynamic model-based method uses smooth CoP transition assumption to split the total forces to each foot, which limited itself to specific movement types, such as normal gaits. We would like to systematically investigate the anterior–posterior and medial–lateral force estimations in a separate study, so that general methods can be developed that do not depend on motion types. On the other hand, only with vertical forces, the established wearable measurement sensor setup supports good joint torque estimations at the ankle joint, when compared with the laboratory-based system.

Another source for the errors found in knee and ankle joint torques can be due to the differences in CoP between the two systems. When calculating joint torques (inverse dynamics) of the wearable system, we aligned the most rear point of pressure insoles with the origin of the Calcaneus bones in the OpenSim Gait2392 model (Delp et al., 2007). This assumption may cause an offset in the CoPs, which in turn causes some errors in computing joint torques. However, since there was no tracking on how feet were placed on the pressure insoles (they are covered by shoes), no better assumptions could be made. This assumption generated quite accurate joint torques, especially at the ankle joint. In addition, the GRFs of the laboratory-based system (the middle column of Figure 8) have been transformed into the calcaneus coordinate frames ( 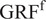 in Figure 2). This enabled easier comparisons of CoPs, since they have the same local coordinate frames as the pressure insoles, no matter where participants stepped on the treadmill and how large the belt speeds were. The vertical force is always perpendicular to the bottom surface of the feet, instead of the treadmill belts. This also provided a better comparison between the two systems, since pressure insoles were in shoes and attached to the bottom of the feet, which is not always parallel with the treadmill surface.

**Figure A1.**
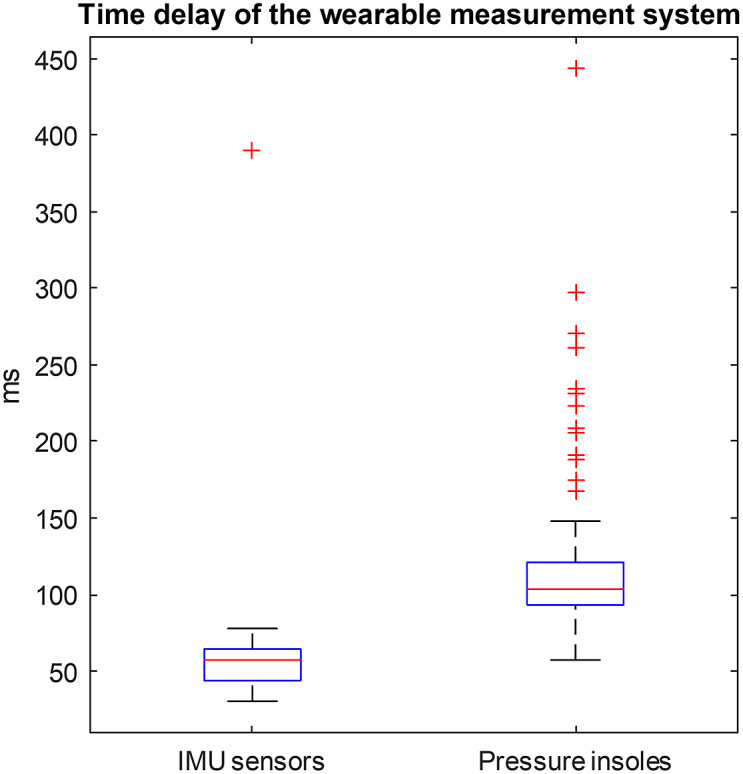
Time delays of the established wearable measurement system. Time delay of all recorded movement trials of all participants is included in this box plot.

The synchronization protocol was established for all sensors that were involved in the experiment recordings. Up to 200 ms delay existed between sensors (Figure A1 in Appendix A), due to the nature of TCP/UDP communications. Offline synchronization was employed to eliminate errors that might have been caused by the data frame offset when calculating joint torques. As a result, the joint torques calculated by the wearable measurement system are the best possible results without considering the data transfer delays. Using the recorded marker data and the instrumented treadmill GRFs as references (their data were accurately aligned because of the analog connections), data streamed from Moticon pressure insoles has an average latency of about 61.3 ± 44.1 ms compared to the Xsens IMUs data. This latency mainly came from two stages of wireless data transfer that Moticon pressure insole used: pressure insole data → (via Bluetooth) → smartphone App → (via WiFi) → computer host. It is possible to reduce the latency by removing the middle step. Another advantage is that the direct communication made it possible to connect two insoles with different sizes for special usage cases. However, this is currently beyond the scope of this work. For the proposed real-time frame-by-frame data streaming setup of the studied wearable measurement system, up to 100 ms latency may exist, which should be considered when establishing and using the same system.

Research on prototyping sensor-level hardware was not included in this study. Commercial sensor products were used to establish the wearable kinetic measurement system. This will enable other research groups who want to achieve the same wearable kinetic measurement capabilities to purchase the sensors and build up the system according to what is described in this paper. Indeed, exploring new technologies on wearable sensors is recommended. However, many wearable measurement systems developed in research laboratories were not thoroughly evaluated, in turn they were not widely used and integrated into other studies from different research groups. Commercial sensor-based systems have the potential to be used by all laboratories and clinical practices around the world once their capabilities and accuracy are independently verified.

One limitation of the current system is the commercial price of the wearable components used. While being less expensive than a laboratory-based system, it is costly for educational purposes or personal use. The creation of low-cost, open-source, and open-hardware device could be beneficial for this. For instance, the much cheaper off-the-shelf IMU chips, however, require development of custom code for drifting compensation. Recently, the open-source biomechanics software OpenSim released the new inverse kinematics function that supports estimating joint angles from raw orientation data of IMU sensors (Al Borno et al., 2022). This can be a promising way to replace the Xsens IMU sensors and its MVN software, and a cheaper wearable measurement system could be established.

Another limit of the current study is that the real-time biomechanical analysis modules (such as the real-time inverse dynamics) were not included. In our long-term study plan, it will be the last step, after improving the wearable measurement setup with respect to the issues discussed above. Considering the wide variety of movement types in human locomotion (such as gait, non-gait daily activities, and sport movements), as well as the limitations of current wearable sensor setup, it is unrealistic to solve such a long-lasting and complex problem within one research paper. Within the best of our knowledge, despite several attempts with specific movement focuses, such as lifting, slow walking, and skiing (Khurelbaatar et al., 2015; Lee et al., 2017; Purevsuren et al., 2018; Conforti et al., 2020; Inertial et al., 2022), there has not been any study that proposed and validated a generic and fully wearable kinetic measurement system. We would like to solve this problem, not in one shot but with a step-by-step plan. Four major steps are planned:

- Step 1: Identify appropriate wearable kinetic measurement sensors and establish real-time frame-by-frame data streaming protocols.
- Step 2: (Offline) Validate the measured kinematics (joint angles), kinetics (forces), and joint kinetics (joint torques) against the current golden-standard conventional system.
- Step 3: Improve the wearable measurement system based on the validation results.
- Step 4: Establish the real-time biomechanical analysis module based on the refined wearable measurement sensor setup.

We believe that these four steps must be taken in a step-by-step manner, to avoid mis-distributing the efforts. For example, without the comprehensive validation of biomechanical analysis (in Step 2), the directions of Step 3 improvement will not be clear. The real-time biomechanical analysis (in Step 4), including inverse dynamics, is not an obstacle. Several studies (Van Den Bogert et al., 2013; Pizzolato et al., 2017; Stanev et al., 2021) have explored it and shown its feasibility. However, without the improvement from Step 3, biomechanical analysis results from the wearable sensor setup are barely useful. For instance, our validation study showed that the data streaming latency of the sensor setup is relatively high. And because of the missing shear forces, joint torque calculations at the knee and hip joints are not accurate compared to the current golden-standard conventional system.

Future work will focus on estimating the shear forces for the wearable measurement system using a generalized method, so that more motion types can be covered. We believe this may require the combination of multi-body dynamic models (Nitschke et al., 2020) and machine learning (Ancillao et al., 2018). In addition, we plan to develop wearable tablet hosts, so that non-portable PC and the external smartphone (in Figure 1) could be replaced by a single fully portable device. With the wearable tablet host, users can freely wear the measurement system in outdoor environments and even at home on a daily basis. As further future step, we would like to implement the real-time joint torque calculation pipeline that we previously developed (Durandau et al., 2018) with the wearable system, so that it can estimate kinetics and muscle-level information in real time. Another future step is to validate the wearable measurement system on patient groups (such as amputee, stroke patients, and patients with spinal cord injury).

## 5. Conclusion

Based on currently available commercial sensors, a wearable kinetic measurement system was established and validated against a conventional laboratory-based setup. Among 13 daily motion types, the wearable system was able to provide accurate joint kinematics, vertical GRFs, as well as torques at the ankle joint. Acceptable matches were achieved for the measured CoPx and the calculated knee joint torques. However, measured mediolateral CoP and calculated hip joint torques did not fit at all with the results of conventional laboratory-based system.

## Figures and Tables

**Figure B1.**
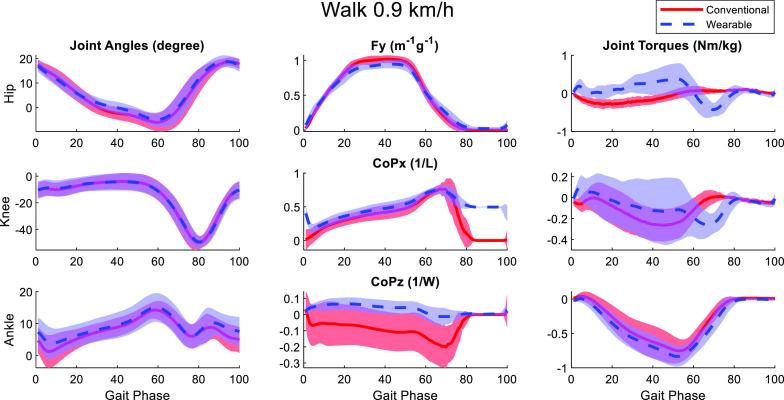
Averaged joint angles (first column), GRFs (second column), and joint torques (third column) in the sagittal plane calculated from the laboratory-based and wearable measurement system from all participants at the walking speed of 0.9 km/h. The Fy, CoPx, and CoPz were transformed into the calcaneus coordinates of the OpenSim model of the participants. Red solid lines and blue dashed lines represent the mean value of these variables from the laboratory-based and wearable systems, respectively. The fading areas indicate one standard deviation of the corresponding variables.

**Figure B2.**
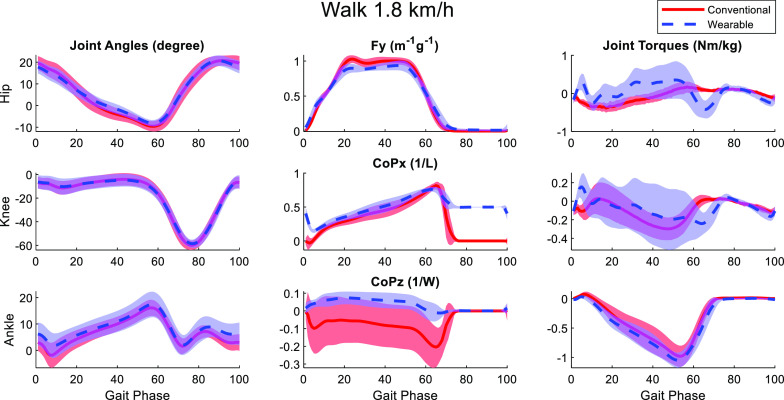
Averaged joint angles (first column), GRFs (second column), and joint torques (third column) in the sagittal plane calculated from the laboratory-based and wearable measurement system from all participants at the walking speed of 1.8 km/h. The Fy, CoPx, and CoPz were transformed into the calcaneus coordinates of the OpenSim model of the participants. Red solid lines and blue dashed lines represent the mean value of these variables from the laboratory-based and wearable systems, respectively. The fading areas indicate one standard deviation of the corresponding variables.

**Figure B3.**
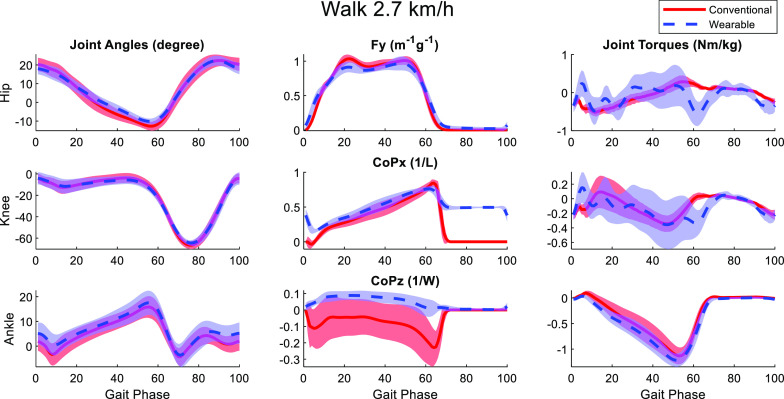
Averaged joint angles (first column), GRFs (second column), and joint torques (third column) in the sagittal plane calculated from the laboratory-based and wearable measurement system from all participants at the walking speed of 2.7 km/h. The Fy, CoPx, and CoPz were transformed into the calcaneus coordinates of the OpenSim model of the participants. Red solid lines and blue dashed lines represent the mean value of these variables from the laboratory-based and wearable systems, respectively. The fading areas indicate one standard deviation of the corresponding variables.

**Figure B4.**
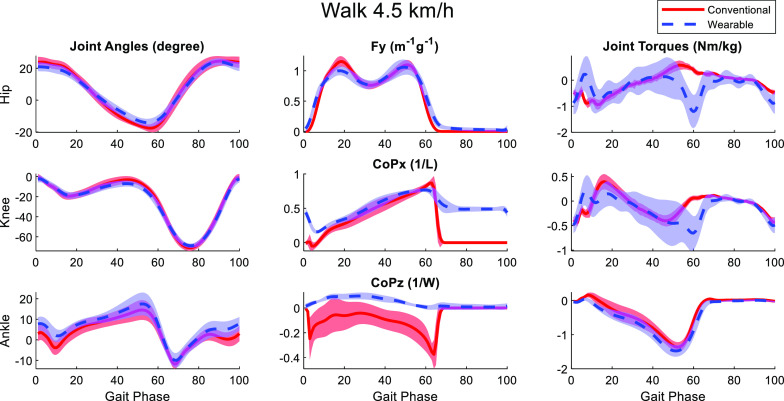
Averaged joint angles (first column), GRFs (second column), and joint torques (third column) in the sagittal plane calculated from the laboratory-based and wearable measurement system from all participants at the walking speed of 4.5 km/h. The Fy, CoPx, and CoPz were transformed into the calcaneus coordinates of the OpenSim model of the participants. Red solid lines and blue dashed lines represent the mean value of these variables from the laboratory-based and wearable systems, respectively. The fading areas indicate one standard deviation of the corresponding variables.

**Figure B5.**
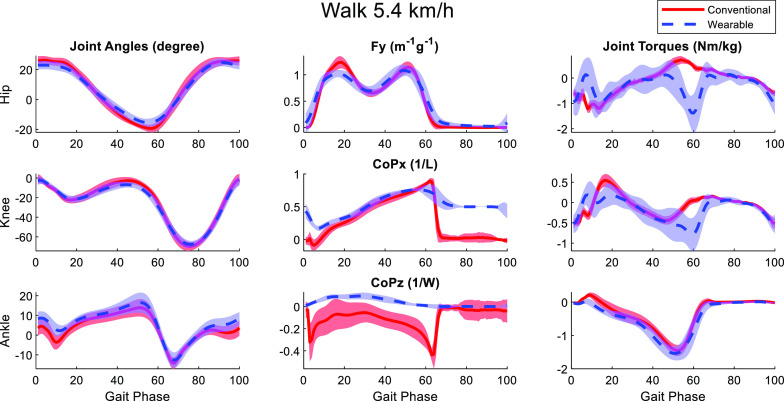
Averaged joint angles (first column), GRFs (second column), and joint torques (third column) in the sagittal plane calculated from the laboratory-based and wearable measurement system from all participants at the walking speed of 5.4 km/h. The Fy, CoPx, and CoPz were transformed into the calcaneus coordinates of the OpenSim model of the participants. Red solid lines and blue dashed lines represent the mean value of these variables from the laboratory-based and wearable systems, respectively. The fading areas indicate one standard deviation of the corresponding variables.

**Figure B6.**
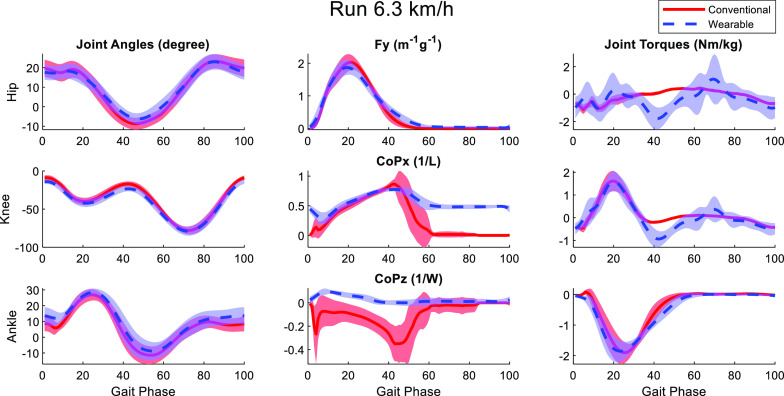
Averaged joint angles (first column), GRFs (second column), and joint torques (third column) in the sagittal plane calculated from the laboratory-based and wearable measurement system from all participants at the running speed of 6.3 km/h. The Fy, CoPx, and CoPz were transformed into the calcaneus coordinates of the OpenSim model of the participants. Red solid lines and blue dashed lines represent the mean value of these variables from the laboratory-based and wearable systems, respectively. The fading areas indicate one standard deviation of the corresponding variables.

**Figure B7.**
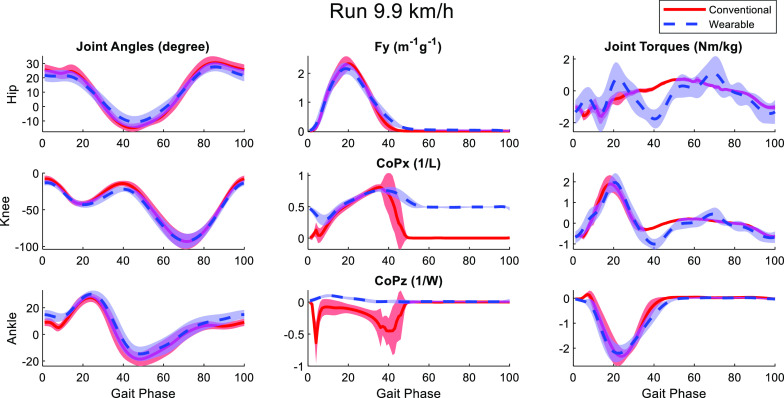
Averaged joint angles (first column), GRFs (second column), and joint torques (third column) in the sagittal plane calculated from the laboratory-based and wearable measurement system from all participants at the running speed of 9.9 km/h. The Fy, CoPx, and CoPz were transformed into the calcaneus coordinates of the OpenSim model of the participants. Red solid lines and blue dashed lines represent the mean value of these variables from the laboratory-based and wearable systems, respectively. The fading areas indicate one standard deviation of the corresponding variables.

**Figure B8.**
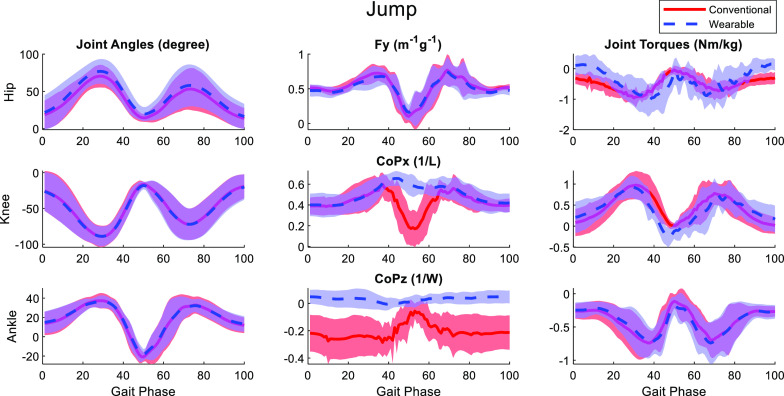
Averaged joint angles (first column), GRFs (second column), and joint torques (third column) in the sagittal plane calculated from the laboratory-based and wearable measurement system from all participants of the jump movements. The Fy, CoPx, and CoPz were transformed into the calcaneus coordinates of the OpenSim model of the participants. Red solid lines and blue dashed lines represent the mean value of these variables from the laboratory-based and wearable systems, respectively. The fading areas indicate one standard deviation of the corresponding variables.

**Figure B9.**
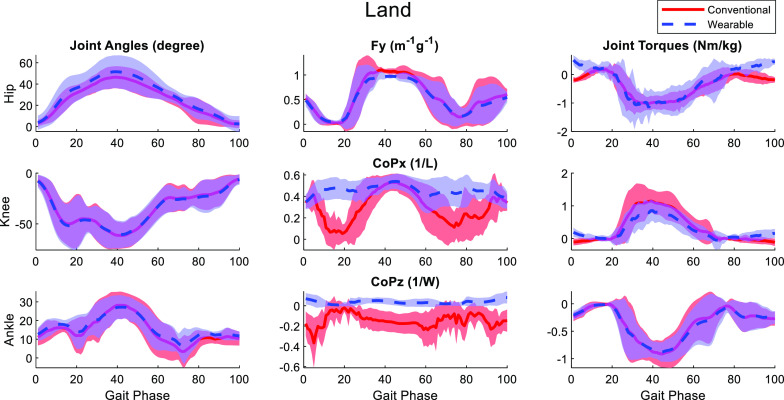
Averaged joint angles (first column), GRFs (second column), and joint torques (third column) in the sagittal plane calculated from the laboratory-based and wearable measurement system from all participants of the single-leg landing movements. The Fy, CoPx, and CoPz were transformed into the calcaneus coordinates of the OpenSim model of the participants. Red solid lines and blue dashed lines represent the mean value of these variables from the laboratory-based and wearable systems, respectively. The fading areas indicate one standard deviation of the corresponding variables.

**Figure B10.**
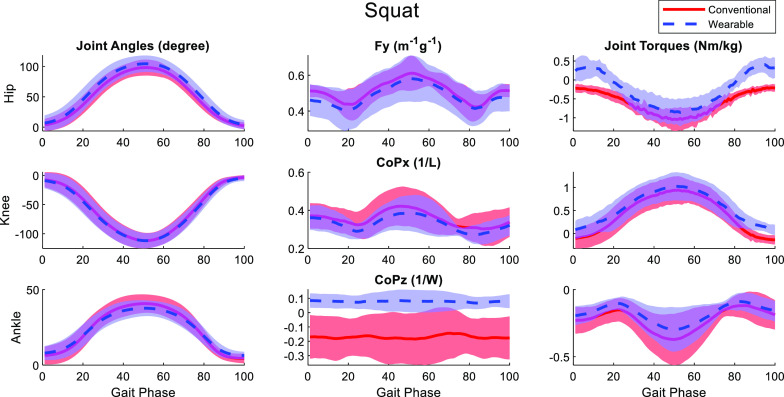
Averaged joint angles (first column), GRFs (second column), and joint torques (third column) in the sagittal plane calculated from the laboratory-based and wearable measurement system from all participants of the squat movements. The Fy, CoPx, and CoPz were transformed into the calcaneus coordinates of the OpenSim model of the participants. Red solid lines and blue dashed lines represent the mean value of these variables from the laboratory-based and wearable systems, respectively. The fading areas indicate one standard deviation of the corresponding variables.

## Data Availability

An open-access dataset (https://doi.org/10.5281/zenodo.6457662) was generated from the experiment providing both raw and processed data of six types of synchronized motion sensors. Documentation, data processing codes, and guidelines to establish the real-time wearable kinetic measurement system are also shared in a public GitHub repository (https://github.com/HuaweiWang/WearableMeasurementSystem).

## Footnotes

1
https://github.com/HuaweiWang/WearableMeasurementSystem.

2
https://doi.org/10.5281/zenodo.6457662.

3
https://github.com/Huawei-UTwente/BioMechPro/tree/study/WearableSystemValidation.

## Appendix A

Time delay of the established wearable measurement system is shown in Figure A1. Time delays of both IMU sensors and pressure insoles were calculated. IMU sensor time delays were calculated between the Xsens IMU lower-limb joint angles and the ones extracted from the optical motion capture system. Pressure insole time delays were calculated between the vertical GRF from Moticon pressure insoles and from the instrumented treadmill.

## Appendix B

Comparison plots between laboratory-based system and the wearable system in other motion trials (Figures B1–B10).
